# Defining the Opioid Requirement in Anterior Cruciate Ligament Reconstruction

**DOI:** 10.5435/JAAOSGlobal-D-21-00298

**Published:** 2022-01-13

**Authors:** Eli T. Sayegh, Tracey S. Otto, Kirsten D. Garvey, Anna Martin, Natalie A. Lowenstein, Elizabeth G. Matzkin

**Affiliations:** From the Department of Orthopaedic Surgery, Brigham and Women's Hospital, Boston, MA (Dr. Sayegh, Dr. Garvey, Lowenstein, and Dr. Matzkin); the Rutgers Robert Wood Johnson Medical School, New Brunswick, NJ (Dr. Otto); and the Trinity College Dublin, Dublin, Ireland (Martin).

## Abstract

**Introduction:**

The amount and duration of opioids necessary after anterior cruciate ligament reconstruction (ACLR) are inadequately defined. This study sought to prospectively (1) define the amount and duration of opioid consumption, (2) investigate the relationship between preoperative pain expectation and postoperative satisfaction with pain management, and (3) identify risk factors for increased opioid use after ACLR.

**Methods:**

One hundred eight patients undergoing primary ACLR with hamstring graft were prospectively analyzed for preoperative pain expectation, using visual analog scale (VAS) rating, and postoperative satisfaction with pain management. Univariate and multivariate analyses were done to identify patient characteristics associated with satisfaction and/or amount and duration of opioid use.

**Results:**

Mean duration and cumulative intake of opioid consumption after ACLR were 5.3 days and 15.3 tablets, respectively. Patients expected moderate postoperative pain: mean preoperative VAS = 68.9. The preoperative VAS rating was associated with a significantly greater amount (*P* = 0.0265) and longer duration (*P* = 0.0212) of opioid consumption. Baseline opioid users took opioids for twice as long postoperatively (10.0 versus 5.0 days; *P* = 0.0149) and consumed twice as many tablets (29.3 versus 14.8 tablets; *P* = 0.0280) compared with opioid-naive patients.

**Discussion:**

This study demonstrated on average 15.3 opioid tablets over 5.3 days provided satisfactory pain management after ACLR. Risk factors for increased opioid consumption included preoperative opioid use.

Oral opioid medications are frequently prescribed for postoperative pain control after common ambulatory orthopaedic procedures. However, their extended use has not only been shown to increase the risk of short-term and long-term medical complications but also the potential for dependency, abuse, and overdose.^1^ The Centers for Disease Control and Prevention estimates that opioid-related complications cost the US economy $78.5 billion annually.^2^ Although the United States represents less than 5% of the global population, Americans consume 80% of the worldwide opioid supply, including 99% of the hydrocodone.^3^ There were 52,000 deaths in the United States in 2015 because of opioid overdose, with 15,000 of those involving a prescribed opioid.^4^ In fact, deaths due to opioid abuse more than quadrupled in 2 decades since 1999.^5^ According to the Centers for Disease Control and Prevention, opioids were involved in 49,680 overdose deaths in 2019, accounting for 70.6% of all drug overdose deaths.^6^ There have been numerous calls to action within the broader medical community for greater stewardship of opioid prescribing and recognition of the hazards they pose to patients.^7,8^ Orthopaedic surgeons, in particular, must be cognizant of the current opioid epidemic and consider actionable ways in which they can stem it in their daily practice. With orthopaedic surgeons ranking the third-highest prescribers across all medical specialties,^9^ the orthopaedic literature^10-12^ has increasingly called for minimizing leftover opioid medication and transitioning to multimodal, primarily non–opioid-based postoperative analgesia regimens. Although the development of standardized institutional protocols has been shown to mitigate opioid overprescription after surgery,^13-15^ there is a paucity of evidence to formulate these for many orthopaedic procedures.

Anterior cruciate ligament reconstruction (ACLR) is one of the most common orthopaedic procedures, with approximately 200,000 operations done in the United States every year.^16,17^ Recent studies have heightened awareness of opioid consumption after arthroscopic knee procedures. Adolescents and young adults undergoing knee arthroscopy and ligament reconstruction or tibial tubercle osteotomy consumed only one-third of the 51 opioid tablets they were on average prescribed, with 11% requiring no opioids at all and only 1% requesting a refill at 3 weeks.^18^ The median amount of opioid medication taken after knee arthroscopy is seven tablets, with over 80% of the patients consuming 20 tablets or less.^19^ ACLR ranked second-highest in opioid prescription duration and highest in prescription refill rate at 7.4 days and 39.3%, respectively, among eight common orthopaedic and general surgical procedures reviewed in a large data repository.^20^ One goal of newer surgical techniques, such as all-inside ACLR, is to reduce pain and postoperative opioid requirements associated with bone-tunnel drilling. A randomized controlled trial of 150 patients comparing all-inside versus full-tunnel ACLR with allograft found no difference in opioid consumption at 1 week (27.8 versus 32.2 oxycodone-acetaminophen-equivalents, respectively). Conversely, in a recent study, median postdischarge opioid consumption after ACLR with autograft (hamstring or patellar bone-tendon-bone) or allograft was only seven tablets, and 71% of the patients had stopped using opioid medications after 1 week,^21^ encouraging rethinking of opioid-prescribing regimens.

The purpose of this prospective study was to define the amount and duration of opioid and nonopioid medications used and assess patient satisfaction 2 weeks after ACLR with a multimodal analgesia regimen incorporating nonopioid medications and a limited opioid prescription. Our expectation was that these findings could alter opioid-prescribing practices and inform the standardized prescribing guidelines. Our specific goals were to (1) assess preoperative patient expectation of postoperative pain, (2) measure postoperative patient satisfaction with their pain management, (3) quantify the typical amount and duration of opioid consumption, and (4) identify whether preoperative expectation or patient characteristics is associated with postoperative satisfaction or opioid consumption. The a priori hypothesis of this study was that greater than 80% of the ACLR patients would be satisfied with a multimodal pain management regimen incorporating a limited opioid taper.

## Methods

### Study Design

From January 2017 to October 2020, a consecutive cohort of 110 adult patients undergoing primary arthroscopic ACLR with hamstring autograft or allograft were prospectively enrolled at a single academic medical center after institutional review board approval. Exclusion criteria included age younger than 18 years, incomplete follow-up data, open approach, bone-patellar tenon-bone graft, quadriceps tendon graft, revision surgery, and/or multiligament surgery. Informed consent was obtained in clinic for patient participation in the study. Participants completed an electronic questionnaire in clinic. Demographic information and patient-reported outcome scores were collected. In addition, the survey included the Patient Health Questionnaire-2 to screen patients for baseline symptoms of depression and the Pain Self-Efficacy Questionnaire-2 to determine the patient's ability to accomplish daily goals and tasks while experiencing pain. Using a 100-point visual analog scale (VAS), the participants were asked to preoperatively rate their anticipated pain 48 hours after surgery. A score of 0 represented no pain, whereas a score of 100 represented the worst possible pain. Patients again rated their pain on the first postoperative day and at the 2-week postoperative visit. Satisfaction with pain management was assessed with two survey questions asking whether patients believed that their pain was (1) well-controlled and (2) properly addressed by hospital staff. The survey queried patients regarding the preoperative use of opioid medications having a strength equivalent to codeine or greater in morphine-equivalent units.

At the 2-week postoperative visit, patients reported opioid and nonopioid consumption for the type of medication used, duration of use, interval and cumulative dose (milligrams), and quantity of tablets used. Satisfaction with pain management was measured on a four-point Likert scale (“never,” “sometimes,” “usually,” or “always”) in response to the following two survey questions: (1) “In the time after surgery, how often was your pain well-controlled?” and (2) “In the time after surgery, how often did the hospital staff do everything they could to help with your pain?” They also completed the Patient Health Questionnaire-2 again at this visit. Patient-reported data were collected in REDCap (Research Electronic Data Capture; Vanderbilt University), which is a browser-based electronic data capture system and subsequently stored in Surgical Outcome System (Arthrex, Naples, FL), which is a Health Information Portability and Accountability Act-compliant global registry database.

### Surgical Technique

All participants received a preoperative ultrasound-guided adductor canal block and an intra-articular injection of 20 cc of 1% bupivacaine without epinephrine at the conclusion of the procedure. All patients underwent arthroscopic ACLR using an all-inside technique with cortical button fixation done by a single surgeon (E.G.M.). Hamstring autograft or allograft was used in all cases. Meniscal and chondral pathology was simultaneously addressed if present. After surgery, all patients were placed into a cryotherapy cuff, constrained knee brace locked in extension with weight-bearing as tolerated and initiated on a standardized, evidence-based postoperative rehabilitation protocol. Postoperatively, patients received nonopioid prescriptions for acetaminophen and a nonsteroidal anti-inflammatory drug, namely, either ibuprofen or naproxen. They were also prescribed a 20-tablet opioid supply of either oxycodone (5 mg) or tramadol (50 mg), without refills, to use for severe pain only if needed.

### Statistical Analysis

Using a convenience sample of 110 subjects, this study was powered to detect between-group differences of approximately 0.54 SD; in other words, if the SD of total tablets of opioid medication was 14, there would be >80% power to detect differences of approximately 7.5 tablets. For continuous variables, this study was powered to detect correlation coefficients of approximately 0.27. Means, SDs, and medians are presented for continuous variables. Number and percent are presented for categorical variables. The associations between continuous outcomes (number of tablets and number of days) and continuous predictors were analyzed with the Spearman correlation coefficient and between continuous outcomes and categorical predictors using the Wilcoxon rank sum test. The associations between categorical outcome (satisfaction) and continuous variables were analyzed using the Wilcoxon rank sum test and between categorical outcome and categorical predictors using the Pearson chi-square test or Fisher exact test. Variables with *P* values less than 0.10 in bivariate analysis were advanced to multivariable models. Owing to the skewed distribution of the number of tablets and the number of days of opioid medication use, we used a square root transformation in the linear regression model. Satisfaction was analyzed with multivariable logistic regression. All *P* values less than 0.05 were considered statistically significant. All statistical analyses were done using SAS version 9.4 (SAS Institute, Cary, NC).

## Results

### Demographic and Clinical Characteristics

A total of 110 patients were prospectively enrolled in the study, of whom 108 patients had complete data (Table 1). Sixty-one patients (56.5%) were female and 47 (43.5%) were male. The mean age was 34.5 ± 12.3 years (range, 18-66 years), and the mean body mass index was 25.7 kg/m^2^ (Table 1). The mean symptom duration was 4.0 ± 5.9 months. One hundred three patients (95.4%) were opioid-naive before surgery. The mean preoperative VAS rating of expected pain 48 hours after ACLR was 68.9 ± 16.4, reflecting an expectation of moderate pain.

**Table 1:** Descriptive Characteristics of Patient Cohort

Variable	N (%)
Total participants	108
Sex	
Female	61 (56.5)
Male	47 (43.5)
Age group	
25-35	37 (34.3)
≤25	27 (25.0)
>35	44 (40.7)
Race	
Asian	13 (12.0)
Black or African American	9 (8.3)
White	86 (79.6)
Are you currently taking any narcotic pain medication (codeine or stronger)?	
No	103 (94.7)
Yes	5 (5.3)

	Mean (SD)
Age	34.5 (12.3)
BMI	25.7 (4.1)
Symptom duration	
During 48 hr after surgery, how do you anticipate your pain to be? (on a scale of zero being no pain and 100 being the worst possible pain)	68.9 (16.4)

### Postoperative Satisfaction with Pain Management

In general, 71.3% of the patients were satisfied (indicating “always” or “usually”) in response to how often pain was well-controlled after ACLR (Table 2). Although there was a trend toward increased satisfaction among female patients relative to their male counterparts (78.7 versus 61.7% “always” or “usually” satisfied; *P* = 0.0530), no demographic or clinical characteristics were associated with postoperative satisfaction with pain management. Notably, preoperative pain expectation was not predictive of postoperative satisfaction, with an average prospective VAS rating of 72.2 in the “sometimes” or “never” satisfied group versus 67.6 in the “always” or “usually” satisfied group (*P* = 0.1984) (Table 3).

**Table 2:** Primary Postoperative Outcomes

Outcome	N (%)
After surgery, did you take any pain medications, such as acetaminophen, ibuprofen, or naproxen?	
No	6 (5.6)
Yes	102 (94.4)
Did you take any narcotic pain medication after your surgical procedure?	
No	8 (7.4)
Yes	100 (92.6)
In the time after surgery, how often was your pain well-controlled?	
Never	1 (0.9)
Sometimes	30 (27.8)
Usually	56 (51.9)
Always	21 (19.4)

	Mean (SD)
Total tablets of narcotic medication	15.3 (13.9)
Days narcotic medication taken	5.3 (4.1)

**Table 3:** Predictors of Satisfaction With Postoperative Pain Control

Characteristic	Never/Sometimes, N = 27	Usually/Always, N = 68	*P*
	Mean (SD)	Mean (SD)	
Age	33.2 (12.2)	35.0 (12.4)	0.4204
BMI	26.3 (5.0)	25.5 (3.6)	0.5558
Symptom duration (months)	3.0 (5.1)	4.4 (6.1)	0.7039
During 48 hr after surgery, how do you anticipate your pain to be? (on a scale of 0 being no pain and 100 being the worst possible pain)	72.2 (11.7)	67.6 (17.9)	0.1984

	N (%)	N (%)	
Age group			0.8199
≤25	12 (39)	25 (32)	
25-35	7 (23)	20 (26)	
>35	12 (39)	32 (42)	
Sex			0.0530
Male	18 (58)	29 (38)	
Female	13 (42)	48 (62)	
Race category			0.3735
White	23 (74)	63 (82)	
Non-White	8 (26)	14 (18)	
Are you currently taking any narcotic pain medication (codeine or stronger)?			0.5675
No	29 (94)	74 (96)	
Yes	2 (6)	3 (4)	

Shown statistics are Spearman correlation (95% confidence interval) for continuous variables and mean (SD) with Wilcoxon rank test *P* value for categorical variables.

### Nonopioid and Opioid Consumption

Most patients used a combination of nonopioid and opioid medications to manage their postoperative pain. One hundred two patients (94.4%) took acetaminophen, ibuprofen, and/or naproxen after surgery. One hundred patients (92.6%) took at least one opioid tablet, whereas eight patients (7.4%) required no opioid medication.

The mean duration of opioid consumption was 5.3 ± 4.1 days with a mean cumulative intake of 15.3 ± 13.9 tablets. Preoperative opioid users consumed twice as many opioid tablets as their opioid-naive counterparts (29.3 versus 14.8 tablets; *P* = 0.0280). Preoperative pain expectation (*P* = 0.0265), age group (*P* = 0.0210, with those aged 25-35 years and those aged >35 years consuming the most and fewest tablets, respectively), and male sex (*P* = 0.0418) were also significantly associated with higher opioid tablet consumption in the univariate analysis, although these were not independently predictive in the multivariate analysis (Table 4). The multivariate analysis further demonstrated higher opioid tablet consumption among those with prior surgery (*P* = 0.0340) and preoperative opioid use (*P* = 0.0480).

**Table 4:** Predictors of Cumulative Opioid Medication Consumption, Measured in Number of Tablets

Characteristic	Associations	Univariate *P*	Multivariable P^*^
Age	−0.06 (−0.25 to 0.13)	0.5129	—
BMI	0.18 (−0.02 to 0.35)	0.0741	0.8004
Symptom duration	−0.15 (−0.33 to 0.04)	0.1206	—
During 48 hr after surgery, how do you anticipate your pain to be? (on a scale of zero being no pain and 100 being the worst possible pain)	0.22 (0.03 to 0.39)	0.0265	0.0566
Age group		0.0210	0.0806
≤25	36 14.8 (16.2); 9.0		
25-35	27 20.2 (11.7); 22.0		
>35	42 12.7 (12.4); 7.0		
Sex		0.0418	0.1678
Male	47 18.9 (16.0); 16.0		
Female	58 12.4 (11.2); 7.0		
Race category		0.1818	—
White	83 16.3 (14.5); 11.0		
Non-White	22 11.6 (10.7); 10.0		
Have you ever had a surgery before?		0.0822	0.0340
No	34 11.9 (11.8); 9.0		
Yes	71 16.9 (14.6); 15.0		
Are you currently taking any narcotic pain medication (codeine or stronger)?		0.0280	0.0480
No	101 14.8 (13.8); 10.0		
Yes	4 29.3 (8.7); 28.5		

* Variables with univariate *P* value less than 0.1 were included in the multivariable model. Shown statistics are Spearman's correlation (95% confidence interval) for continuous variables and mean (SD) with the Wilcoxon rank test for categorical variables.

Preoperative pain expectation (*P* = 0.0212) and preoperative opioid use (*P* = 0.0149) were each significantly associated with a longer duration of opioid consumption (Table 5) and remained significant in the multivariate analysis (*P* = 0.0414 and *P* = 0.0110, respectively). Baseline opioid users remained on opioids for twice as long after surgery compared with those who were opioid-naive (10.0 versus 5.0 days).

**Table 5:** Predictors of Duration of Opioid Medication Consumption, Measured in Days

Characteristic	Associations	Univariate *P*	Multivariable P^*^
Age	−0.01 (−0.19 to 0.18)	0.9540	—
BMI	0.12 (−0.07 to 0.30)	0.2212	—
Symptom duration	−0.08 (−0.27 to 0.11)	0.4002	—
During 48 hr after surgery, how do you anticipate your pain to be? (on a scale of zero being no pain and 100 being the worst possible pain)	0.22 (0.03 to 0.39)	0.0212	0.0414
Age group		0.5474	—
≤25	37 4.9 (3.4); 4.0		
25-35	27 5.9 (4.1); 5.0		
>35	44 5.2 (4.6); 3.0		
Sex		0.8445	—
Male	47 5.4 (4.1); 4.0		
Female	61 5.2 (4.1); 4.0		
Race category		0.8122	—
White	86 5.2 (4.1); 4.0		
Non-White	22 5.4 (4.0); 5.0		
Have you ever had a surgery before?		0.3379	—
No	34 4.5 (3.3); 3.0		
Yes	74 5.6 (4.4); 4.0		
Are you currently taking any narcotic pain medication (codeine or stronger)?		0.0149	0.0110
No	103 5.0 (4.0); 4.0		
Yes	5 10.0 (4.1); 10.0		

* Shown statistics are Spearman's correlation (95% confidence interval) for continuous variables and mean (SD) with the Wilcoxon rank test *P* value for categorical variables.

## Discussion

Orthopaedic surgeons performing ACLR are responsible for devising a postoperative analgesia regimen that is satisfactory to patients, minimizes both short-term and long-term morbidity, and is rooted in evidence. Given the substantial economic and public health costs associated with opioid abuse, there is an imminent need to define opioid requirements for common procedures, such as ACLR and guide opioid-prescribing practices. Such data validate postoperative analgesia regimens that rely more heavily on nonopioid medications while providing a limited opioid taper. This study sought to prospectively (1) define the required amount and duration of opioid consumption, (2) investigate the relationship between preoperative pain expectation and postoperative satisfaction with pain management, and (3) identify risk factors for increased opioid use over a 2-week follow-up period in a cohort of patients undergoing ACLR.

This prospective study defined a mean opioid requirement of 15.3 tablets over a mean duration of 5.3 days for patients undergoing primary all-inside ACLR with hamstring autograft or allograft. Patients typically expected moderate postoperative pain, with a mean preoperative VAS score of 68.9. Although preoperative VAS scores had no bearing on postoperative patient satisfaction, they were markedly associated with greater amount and longer duration of opioid consumption. We also found that baseline opioid users remained on opioids for twice as long after surgery and consumed twice as many opioid tablets than their opioid-naive counterparts. Additional risk factors for prolonged duration or increased amount of opioid consumption included age group (with those aged 25-35 years and those aged >35 years consuming the most and fewest tablets, respectively), male sex, and prior surgery.

Preoperative opioid use has previously been shown to confer a greater than sixfold risk of filling an opioid medication prescription 1 year after ACLR.^22^ Preoperative opioid use exists in two-thirds of the patients undergoing orthopaedic surgery, which is more pervasive than in any other surgical subspecialty.^23^ It has been previously associated with higher postoperative opioid consumption in patients undergoing knee arthroscopy for meniscal repair, partial meniscectomy, débridement, chondroplasty, or loose body removal.^19^ Recently, preoperative opioid use was found to predict markedly prolonged postoperative opioid use and poorer functional outcome scores 6 and 12 months after ACLR.^24^

The results of this study have the potential to change clinical practice, minimize the retention and/or circulation of unused opioid tablets, and reduce downstream complications and healthcare costs. They can guide the formulation of procedure-specific opioid-prescribing guidelines, potentially stemming the wave of opioid overconsumption and misuse. They also serve as an impetus for further, similarly designed research studies that aim to define opioid requirements for common outpatient procedures. Our findings also highlight the need for patient-targeted counseling, education, and/or cognitive-behavioral interventions before surgery to gauge, assuage, and, in some cases, redirect patient expectations about the nature and severity of pain they expect to experience after surgery.

Our findings are clinically applicable in light of building evidence that preoperative opioid education and expectation-setting can markedly reduce postoperative opioid consumption. A randomized controlled trial of patients undergoing arthroscopic rotator cuff repair at the Rothman Institute recently assessed the efficacy of a formal preoperative education program, which described recommended postoperative opioid use, adverse events, and potential for addiction and dependence.^25^ The study group consumed markedly less opioid medication at 6 and 12 weeks, and baseline opioid users were 6.8 times more likely to stop using opioids. Other studies have shown preoperative counseling, modifications to default electronic medical record order sets, and/or pocket reminder cards for clinicians markedly reduce postoperative opioid use after orthopaedic procedures.^26-28^ As far as prescribers are concerned, multiple studies have shown that relatively few patients recovering from knee or shoulder arthroscopy request a refill after completing their initial prescription.^19,21,29^ Therefore, prescribers should err on the side of minimizing unused tablets because this is not likely to generate a high number of refill requests. Our opioid usage data are consistent with a recent recommendation for a maximum of 20 opioid tablets for 1 week for patients undergoing ACLR.^21^

There are noteworthy limitations to this study. Our study solely included the use of hamstring autografts or allografts, and it is conceivable that patients undergoing autograft harvest with a bone plug might have greater postoperative opioid requirements. Data such as amount and duration of opioid consumption were patient-reported and subject to reporting or recall biases, although the latter is minimized given that data were collected 1 day and 2 weeks after surgery. The Hawthorne effect, which dictates that study participant behavior may be altered because of the awareness of being observed, was minimized by electronically collecting bulk data for multiple arms of this prospective study without specific identification of the study hypothesis. The follow-up period was short-term by design, to define early postoperative opioid requirements, but cannot necessarily be extrapolated to later-term pain management as rehabilitation progresses. Although the identification of independent risk factors for increased opioid use was not the chief goal of this study, our multivariate analysis was underpowered to draw meaningful conclusions.^30^ Incorporation of patient characteristics may eventually allow for individualized prescribing based on predictive modeling.

Strengths of this study include its prospective design, 100% follow-up rate, control of the prescription amount, and standardized technical characteristics, including regional anesthesia, graft and fixation technique, intraoperative anesthetic injection, rehabilitation protocol, and oral analgesic regimen, improving the reliability of our findings and limiting the influence of confounding factors. Furthermore, our interpretation of postoperative pain in the context of a preoperative baseline, which may markedly vary between individuals, achieves normalization and minimizes selection bias.^31^

In summary, pain after ACLR can be effectively controlled with a multimodal analgesia regimen incorporating a limited opioid taper of 15 tablets for 5 days, without compromising patient satisfaction. Enumeration of the opioid requirement by well-designed research studies, and increased recognition by both clinicians and patients, is critical for establishing and enacting sound procedure-specific opioid-prescribing guidelines for ACLR. Preoperative interventions involving patient education and expectation-setting should be targeted to at-risk groups. These include patients with heightened preoperative pain expectation and baseline opioid users, the latter of whom consume markedly more opioid medication for a markedly longer duration after ACLR than opioid-naive patients.

In conclusion, this study demonstrated that, on average, 15.3 opioid tablets over a mean of 5.3 days provided satisfactory pain management after ACLR. Risk factors for increased opioid consumption included preoperative opioid use (http://links.lww.com/JG9/A183).

## References

[1] Tabler NG Jr: Over-reliance on opioids poses quality and cost concerns. Heal Financ Manag 2016;70:40-45. 29901345

[2] Maiese BA Pham AT Shah MV Eaddy MT Lunacsek OE Wan GJ: Hospitalization costs for patients undergoing orthopedic surgery treated with intravenous acetaminophen (IV-APAP) plus other IV analgesics or IV opioid monotherapy for postoperative pain. Adv Ther 2017;34:421-435. 27943118 10.1007/s12325-016-0449-8 PMC5331089

[3] Manchikanti L Fellows B Ailinani H Pampati V: Therapeutic use, abuse, and nonmedical use of opioids: A ten-year perspective. Pain Physician 2010;13:401-435. 20859312

[4] Guy GP Zhang K Bohm MK : Vital signs: Changes in opioid prescribing in the United States, 2006-2015. MMWR Morb Mortal Wkly Rep 2017;66:697-704. 28683056 10.15585/mmwr.mm6626a4 PMC5726238

[5] Centers for Disease Control and Prevention: Multiple cause of death data. https://wonder.cdc.gov/mcd.html. Accessed December 21, 2020.

[6] Centers for Disease Control and Prevention: Drug overdose deaths. https://www.cdc.gov/drugoverdose/deaths/index.html. Accessed December 21, 2020.

[7] McCarthy M: Doctors are urged to limit opioid prescribing. BMJ 2016;352:i1614. 26993363 10.1136/bmj.i1614

[8] Blendon RJ Benson JM: The public and the opioid-abuse epidemic. N Engl J Med 2018;378:407-411. 29298128 10.1056/NEJMp1714529

[9] Volkow ND McLellan TA Cotto JH Karithanom M Weiss SR: Characteristics of opioid prescriptions in 2009. JAMA 2011;305:1299-1301. 21467282 10.1001/jama.2011.401 PMC3187622

[10] Leopold SS Beadling L: Editorial: The opioid epidemic and orthopaedic surgery-no pain, who gains? Clin Orthop Relat Res 2017;475:2351-2354. 28762150 10.1007/s11999-017-5454-y PMC5599414

[11] Labrum JT IV Ilyas AM: The opioid epidemic: Postoperative pain management strategies in orthopaedics. JBJS Rev 2017;5:e14. 28857933 10.2106/JBJS.RVW.16.00124

[12] Mir HR Miller AN Obremskey WT Jahangir AA Hsu JR: Confronting the opioid crisis: Practical pain management and strategies: AOA 2018 critical issues symposium. J Bone Joint Surg Am 2019;101:e126. 31800430 10.2106/JBJS.19.00285

[13] Lovecchio F Stepan JG Premkumar A : An institutional intervention to modify opioid prescribing practices after lumbar spine surgery. J Neurosurg Spine 2019:1-8. 10.3171/2018.8.SPINE18386 30738410

[14] Stepan JG Sacks HA Lovecchio FC : Opioid prescriber education and guidelines for ambulatory upper-extremity surgery: Evaluation of an institutional protocol. J Hand Surg Am 2019;44:129-136. 30033347 10.1016/j.jhsa.2018.06.014

[15] Stepan JG Lovecchio FC Premkumar A : Development of an institutional opioid prescriber education program and opioid-prescribing guidelines: Impact on prescribing practices. J Bone Joint Surg Am 2019;101:5-13. 30601411 10.2106/JBJS.17.01645

[16] Davarinos N O'Neill BJ Curtin W: A brief history of anterior cruciate ligament reconstruction. Adv Orthop Surg 2014;2014:1-6.

[17] Evans J Nielson Jl: Knee Cruciate Ligament Anterior Injury. StatPearls Publishing, 2018. 29763023

[18] Tepolt FA Bido J Burgess S Micheli LJ Kocher MS: Opioid overprescription after knee arthroscopy and related surgery in adolescents and young adults. Arthroscopy 2018;34:3236-3243. 30396797 10.1016/j.arthro.2018.07.021

[19] Wojahn RD Bogunovic L Brophy RH : Opioid consumption after knee arthroscopy. J Bone Joint Surg Am 2018;100:1629-1636. 30277992 10.2106/JBJS.18.00049

[20] Scully RE Schoenfeld AJ Jiang W : Defining optimal length of opioid pain medication prescription after common surgical procedures. JAMA Surg 2018;153:37-43. 28973092 10.1001/jamasurg.2017.3132 PMC5833616

[21] Lovecchio F Premkumar A Uppstrom T : Opioid consumption after arthroscopic meniscal procedures and anterior cruciate ligament reconstruction. Orthop J Sports Med 2020;8:2325967120913549. 32426402 10.1177/2325967120913549 PMC7219018

[22] Anthony CA Westermann RW Bedard N : Opioid demand before and after anterior cruciate ligament reconstruction. Am J Sports Med 2017;45:3098-3103. 28806097 10.1177/0363546517719226

[23] Hilliard PE Waljee J Moser S : Prevalence of preoperative opioid use and characteristics associated with opioid use among patients presenting for surgery. JAMA Surg 2018;153:929-937. 29998303 10.1001/jamasurg.2018.2102 PMC6233789

[24] Forlenza EM Lavoie-Gagne O Lu Y : Preoperative opioid use predicts prolonged postoperative opioid use and inferior patient outcomes following anterior cruciate ligament reconstruction. Arthroscopy 2020;36:2681-2688.e1. 32574617 10.1016/j.arthro.2020.06.014

[25] Syed UAM Aleem AW Wowkanech C : Neer award 2018: The effect of preoperative education on opioid consumption in patients undergoing arthroscopic rotator cuff repair: A prospective, randomized clinical trial. J Shoulder Elbow Surg 2018;27:962-967. 29599038 10.1016/j.jse.2018.02.039

[26] Holman JE Stoddard GJ Horwitz DS Higgins TF: The effect of preoperative counseling on duration of postoperative opiate use in orthopaedic trauma surgery: A surgeon-based comparative cohort study. J Orthop Trauma 2014;28:502-506. 24667804 10.1097/BOT.0000000000000085

[27] Alter TH Ilyas AM: A prospective randomized study analyzing preoperative opioid counseling in pain management after carpal tunnel release surgery. J Hand Surg Am 2017;42:810-815. 28890331 10.1016/j.jhsa.2017.07.003

[28] Stanek JJ Renslow MA Kalliainen LK: The effect of an educational program on opioid prescription patterns in hand surgery: A quality improvement program. J Hand Surg Am 2015;40:341-346. 25542435 10.1016/j.jhsa.2014.10.054

[29] Kumar K Gulotta LV Dines JS : Unused opioid pills after outpatient shoulder surgeries given current perioperative prescribing habits. Am J Sports Med 2017;45:636-641. 28182507 10.1177/0363546517693665

[30] Peduzzi P Concato J Kemper E Holford TR Feinstein AR: A simulation study of the number of events per variable in logistic regression analysis. J Clin Epidemiol 1996;49:1373-1379. 8970487 10.1016/s0895-4356(96)00236-3

[31] Farrar JT: Advances in clinical research methodology for pain clinical trials. Nat Med 2010;16:1284-1293. 20948532 10.1038/nm.2249

